# ENHANCING DIVERSITY, EQUITY, AND INCLUSION IN QUANTITATIVE STUDIES OF AGE AND LIFE COURSE

**DOI:** 10.1093/geroni/igae098.1554

**Published:** 2024-12-31

**Authors:** Jessica Kelley

**Affiliations:** Case Western Reserve University, Cleveland, Ohio, United States

## Abstract

Despite best intentions to explore and represent the wide diversity of older adults in research, many are limited in their inquiry when utilizing secondary data sources to the the original investigators’ choices of measurement or sample inclusion. Limitations among these studies regularly include insufficient sample sizes of small groups, low statistical power, or over-aggregation in measurement. Using race/ethnicity for our examples, we highlight two key barriers to DEI initiatives in secondary data analyses and their consequences in studying aging and older adults: (1) Erasure of significant populations in aging research; and (2) Utilizing statistical logic to explain social realities. We then provide five immediate solutions that investigators can execute with the data at hand to increase diversity in aging research. These include greater attention to within-group variability, avoid over-interpreting small differences between groups, incorporating cohort membership and structural inequality into analysis, and integrating new measurement modules into ongoing studies.

